# Creatine Supply Attenuates Ischemia-Reperfusion Injury in Lung Transplantation in Rats

**DOI:** 10.3390/nu12092765

**Published:** 2020-09-10

**Authors:** Francine M. Almeida, Angela S. Battochio, João P. Napoli, Katiusa A. Alves, Grace S. Balbin, Manoel Oliveira-Junior, Henrique T. Moriya, Paulo M. Pego-Fernandes, Rodolfo P. Vieira, Rogerio Pazetti

**Affiliations:** 1 Instituto do Coraçao, Hospital das Clinicas HCFMUSP, Faculdade de Medicina, Universidade de Sao Paulo, Sao Paulo 05508-060, Brazil; francinealmeida@alumni.usp.br (F.M.A.); angela.sbattochio@gmail.com (A.S.B.); paulopego@incor.usp.br (P.M.P.-F.); 2 Laboratorio de Pesquisa em Cirurgia Toracica-LIM61, Hospital das Clinicas HCFMUSP, Faculdade de Medicina, Universidade de Sao Paulo, Sao Paulo 05508-060, Brazil; joaovitorpithon@hotmail.com (J.P.N.); katiusa_alves23@hotmail.com (K.A.A.); grace.balbin@hotmail.com (G.S.B.); 3 Brazilian Institute of Teaching and Research in Pulmonary and Exercise Immunology (IBEPIPE), Sao Jose dos Campos 04372-020, Brazil; manoel.junior@hotmail.com (M.O.-J.); rodrelena@yahoo.com.br (R.P.V.); 4 Biomedical Engineering Laboratory-LEB, University of Sao Paulo, Sao Paulo 05508-060, Brazil; htmoriya@leb.usp.br; 5 Post-Graduation Program in Bioengineering, Universidade Brasil, Sao Paulo 05403-000, Brazil; 6 Post-Graduation Program in Sciences of Human Movement and Rehabilitation, Federal University of Sao Paulo (UNIFESP), Santos 04021-001, Brazil

**Keywords:** lung transplantation, ischemia and reperfusion injury, creatine, inflammation, Sprague Dawley rat

## Abstract

Ischemia-reperfusion injury (IRI) is one of the factors limiting the success of lung transplantation (LTx). IRI increases death risk after transplantation through innate immune system activation and inflammation induction. Some studies have shown that creatine (Cr) protects tissues from ischemic damage by its antioxidant action. We evaluated the effects of Cr supplementation on IRI after unilateral LTx in rats. Sixty-four rats were divided into four groups: water + 90 min of ischemia; Cr + 90 min of ischemia; water + 180 min of ischemia; and Cr + 180 min of ischemia. Donor animals received oral Cr supplementation (0.5 g/kg/day) or vehicle (water) for five days prior to LTx. The left lung was exposed to cold ischemia for 90 or 180 min, followed by reperfusion for 2 h. We evaluated the ventilatory mechanics and inflammatory responses of the graft. Cr-treated animals showed a significant decrease in exhaled nitric oxide levels and inflammatory cells in blood, bronchoalveolar lavage fluid and lung tissue. Moreover, edema, cell proliferation and apoptosis in lung parenchyma were reduced in Cr groups. Finally, TLR-4, IL-6 and CINC-1 levels were lower in Cr-treated animals. We concluded that Cr caused a significant decrease in the majority of inflammation parameters evaluated and had a protective effect on the IRI after LTx in rats.

## 1. Introduction

Lung transplantation (LTx) is a well-established therapeutic option for the treatment of several diseases, such as chronic obstructive pulmonary disease, pulmonary fibrosis, bronchiectasis, and primary pulmonary hypertension [1,2,3]. The major cause of post-transplant death during the first 30 days is graft failure. Other causes include multiple organ and cardiovascular failure and technical failures related to the transplant procedures, as well as bronchiolitis obliterans syndrome [3]. The success of LTx is limited by high rates of primary graft dysfunction (PGD) due to ischemia-reperfusion injury (IRI), which is characterized by inflammation, alveolar damage, and vascular permeability [4].

In LTx, organ ischemia and subsequent reperfusion are inevitable and generally lead to acute and sterile inflammation after transplantation, which is called IRI. There are no therapeutic agents used clinically to prevent IRI, and treatment strategies are limited to support care [4]. However, antioxidant agents have been tested in different experimental models [5,6] including creatine [7,8].

Cr is very important in maintaining and managing cellular ATP reserves in both physiological and pathological states. Besides these ergogenic actions, it has many additional pleiotropic effects, e.g., antioxidant activity. The major therapeutic potential of CR is noted in diseases caused by insufficient energy or increased energy demand, for example, ischemic stroke and other cerebrovascular diseases [9]. Wyss and Kaddurah-Daouk [10] showed that phosphocreatine protects tissues from ischemic damage and may, therefore, have an impact on organ transplantation. Almeida et al. [8] used an animal model of total occlusion of the left pulmonary hilum (artery, vein, and bronchus) to verify the anti-inflammatory and antioxidant effects of Cr monohydrate in attenuating the effects of IRI on pulmonary tissue. We hypothesized that Cr supplementation also has beneficial effects on IRI attenuation in LTx, a more complex model that is closer to clinical surgical practice.

## 2. Materials and Methods

### 2.1. Animals

Sixty-four Sprague Dawley adult male rats (400 g) were provided by the Central Animal Facility of the University of Sao Paulo and maintained at standard conditions according to National Institutes for Health Guide for the Care and Use of Laboratory Animals. This study was approved by the Research Ethics Committee of the Faculty of Medicine, University of Sao Paulo (CEP 376/13).

### 2.2. Groups

**Figure 1:**
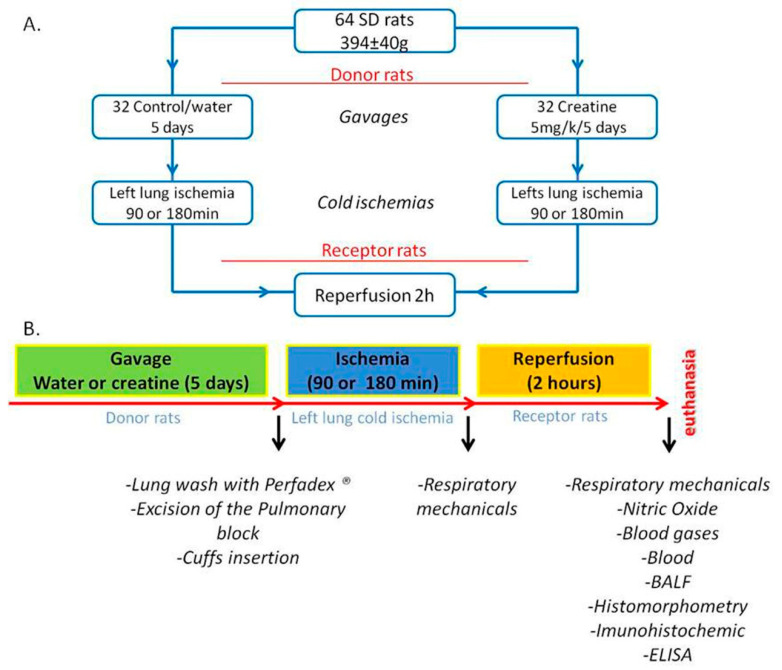
Study design. (**A**) groups and treatment; (**B**) methods and analysis.

Receptors animals were divided into four groups according to donor treatment: (i) donor supplementation with water/vehicle or Cr monohydrate; and (ii) cold ischemia time for 90 or 180 min (Figure 1A).

### 2.3. Creatine Supplementation

Donor animals received Cr (Sigma, 0.5 g/kg/day) diluted in 1000 µL of water by gavage for 5 days before LTx [8,11,12].

### 2.4. Surgical Procedures

Donor animals: twenty-four hours after the last gavage, donors were anesthetized with isoflurane 5% (Isothane, Baxter), orotracheally intubated, and mechanically ventilated (FlexiVent, SCIREQ, Montreal, CA) with 10 mL/kg, 90 cycles/min, and PEEP 3 cm H2O. General anesthesia during surgical procedures was maintained with isoflurane (2%). After median laparotomy, 50 IU of heparin was injected into the inferior vena cava. Then, median sternotomy was performed, and the pulmonary artery was cannulated for anterograde perfusion with 20 mL of low-potassium dextran (LPD) solution (Perfadex, Vitrolife, Sweden) at 4 °C with constant pressure (20 cm H2O) [13]. Before perfusion, the inferior vena cava was sectioned to decrease venous return, and the left atrial appendage was amputated to drain the LPD. Animals were euthanized by exsanguination. After perfusion, the cardiopulmonary block was excised and kept inflated by tracheal occlusion at the end of inspiration. The cardiopulmonary block was allocated in a moistened petri plate with Perfadex solution at 4 °C, and the left hilum was dissected and cuffs were fixed in the artery, vein, and bronchus, as previously described [14]. The grafts were kept inflated during cold ischemia for 90 or 180 min.

Receptor animals: anesthesia, intubation, and ventilation were performed in the same way as in the donors. Left thoracotomy at the fifth intercostal space was performed, and the left hilum was dissected and clamped as proximal as possible using a stereomicroscope (Model SZ61, Olympus, Tokyo, Japan). Then, graft implantation was performed by introducing graft cuffs into a small hole made in the ventral wall of the artery, vein, and bronchus. After cuff fixation using a 7.0 polypropylene silk, the clamps of the bronchus, vein, and artery were removed to re-establish airflow and circulation in the graft. After surgery, the animals received analgesia (dipyrone 400 mg/kg) through the orogastric duct. Closure of the receptor incision was performed using 2.0 monofilament nylon sutures. The period of reperfusion was 120 min for all animals.

All procedures are summarized in Figure 1B.

### 2.5. Respiratory Mechanics

After immediate reperfusion (re-established airflow and circulation) and 120 min of reperfusion of the graft, impedance of the respiratory system of the animals was calculated by the forced oscillation model [15]. We used airway resistance (RAW), tissue elastance (HTIS), and tissue damping (GTIS) parameters.

### 2.6. Exhaled Nitric Oxide (NOex)

After respiratory mechanics, anesthesia was closed, and ventilation was performed using only ambient air. After 5 min, a mylar balloon was connected to the FlexiVent expiratory way, and NOex was collected for 3 min [8,13]. The NOex concentrations were measured by chemiluminescence using a fast-responding analyzer (NOA 280, Sievers Instruments, Inc., Boulder, CO, USA).

### 2.7. Blood Gases

After NOex sample collection, median laparotomy was performed to collect blood by puncturing the abdominal aorta with a heparinized syringe. Analysis of the blood samples was performed on the Stat Profile 10 apparatus.

### 2.8. Peripheral Blood Cell Count and Euthanasia

Five milliliters of blood from the inferior vena cava were collected to determine the total leukocyte number and differential leukocyte count (200 cells/slide). Blood was centrifuged, and creatinine levels were measured in plasma. Subsequently, the animal was euthanized by abdominal aorta artery section, and the cardiopulmonary block was excised.

### 2.9. Bronchoalveolar Lavage Fluid (BALF) and Inflammatory Mediators

BALF was selectively performed on the left lung by instillation of 5 mL of saline solution. We evaluated the total and differential number of inflammatory cells [16]. The supernatant of BALF was used to measure the levels of IL-6, IL-10, TNF-α, and CINC-1 by ELISA (RD Systems, CA, USA).

### 2.10. Histomorphometric and Immunohistochemistry Study

The left lobe was kept in paraformaldehyde solution for 24 h. After that, it was included in paraffin and slides were prepared with 5 µm thickness sections. The slides were stained with hematoxylin-eosin for the analysis of the density of mono/polymorphonuclear cells in the lung parenchyma compartment and for the perivascular edema index [8,13,16]. The lung parenchyma corresponds to lung areas without airways, constituted by alveoli and alveolar saci and pulmonary vessels, including capillaries. For analysis of the perivascular edema index, the area considered was between the external border of the vascular smooth muscle until the adventitia of the vessel. For immunohistochemistry, histological sections were incubated with anti-PCNA (Dako M0879, 1:50), anti-Caspase-3 (NB500-210, 1:200), anti-TLR-4 (SC30002, 1:100), and anti-TLR-7 (SC30004, 1:25); point-counting histomorphometry was applied to quantify inflammatory cells (macrophages as representant of mononuclear cells and neutrophils as representant of polymorphonuclear cells) in the lung parenchyma.

### 2.11. Statistical Analysis

The normal distribution and homogeneity of variances were evaluated with the Shapiro-Wilk and Levene tests, respectively. We used the Student’s t-test or Mann–Whitney Rank test according to the normality of data distribution, and the statistical analysis was performed using SigmaPlot 11 statistical software. Statistical significance was defined as *p*-value < 0.05.

## 3. Results

Eight animals per group were evaluated. The mean weight of the recipient animals was 394 ± 40 g, while the lung and heart weights were 3081 ± 599 mg and 1208 ± 104 mg, respectively. There was no difference between groups (Table 1).

### 3.1. Respiratory Mechanics

Lung mechanics data were evaluated at the beginning of reperfusion (immediate reperfusion) and after 2 h (final reperfusion). There was an increase in RAW and a decrease in GTIS and HTIS in animals treated with Cr in the immediate reperfusion. However, there was no change in RAW in the final reperfusion (Table 2). Data from donor rats were collected prior to exsanguination (Appendix Table A1).

### 3.2. NOex Concentration

**Figure 2:**
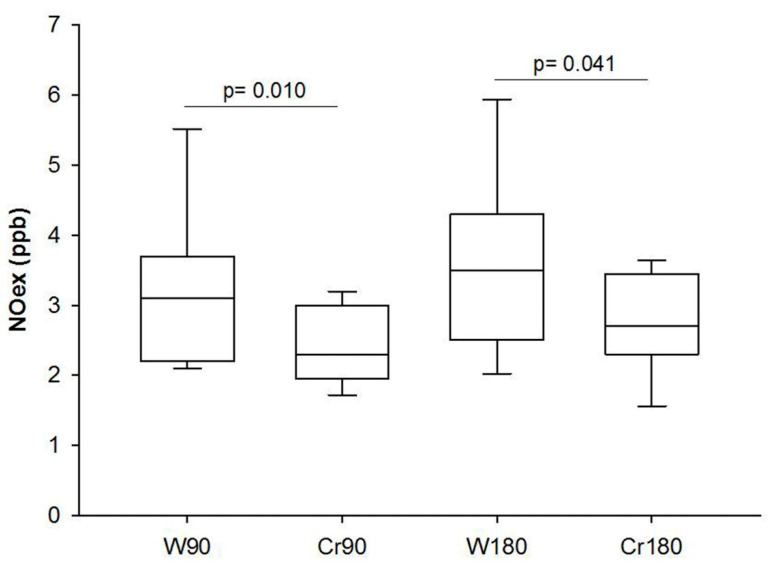
Exhaled nitric oxide (parts per billion). W90, control/water + 90 min of ischemia; Cr90, creatine + 90 min of ischemia; W180, control/water + 180 min of ischemia; Cr180, creatine + 180 min of ischemia. Data are expressed as median and interquartile range. W90 vs. Cr90 and W180 vs. Cr180 (*n* = 8 animals/group).

The creatine-treated animals showed a lower NOex concentration in both ischemia times (Figure 2).

### 3.3. Creatinine and Blood Gas Concentration

There was an increase in plasma creatinine concentration in creatine-treated animals in the two evaluated ischemia times. There was an improvement in the oxygenation of creatine-treated animals after 90 min of ischemia, with a decrease in pCO2 and increase in pO2. There was no difference in lactate concentration (Table 3). The creatinine concentration in donor rats was measured prior to exsanguination (Appendix Table A1).

### 3.4. Inflammatory Cells in the Peripheral Blood and BALF

There was a decrease in the total number of leukocytes, neutrophils, and monocytes/macrophages counted in the blood smears and BALF of creatine-treated animals. There was a difference in the number of lymphocytes in the blood smears only in creatine-treated animals after 180 min of ischemia (Table 4).

### 3.5. Lung Parenchyma Inflammation and Edema Index

**Figure 3:**
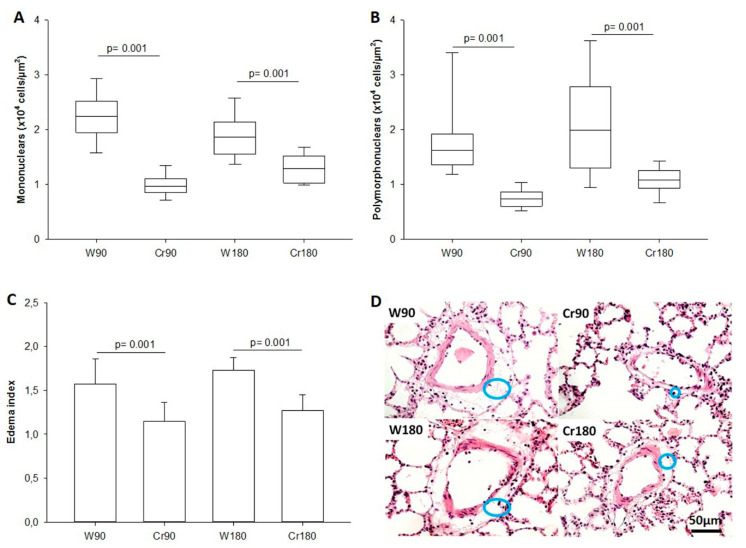
Inflammation and edema index in the lung parenchyma. W90, control/water + 90 min of ischemia; Cr90, creatine + 90 min of ischemia; W180, control/water + 180 min of ischemia; Cr180, creatine + 180 min of ischemia. (**A**) Mononuclear cells; (**B**) polymorphonuclear cells; (**C**) perivascular edema index; (**D**) photomicrographs of the perivascular regions: blue circles show perivascular edema area (H.E., 400×). Data are expressed as median and interquartile range (**A**,**B**) or mean and standard deviation (**C**) (*n*= 8 animals/group).

**Figure 4:**
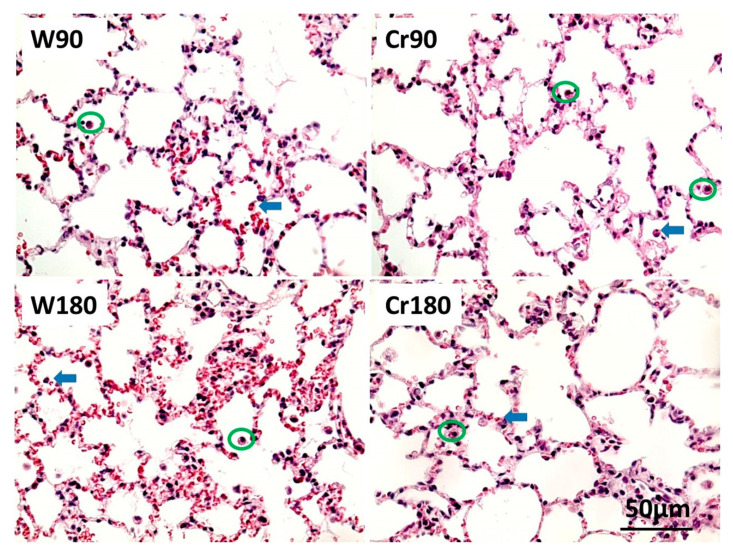
Mononuclear and polymorphonuclear cells in the lung tissue. W90, control/water + 90 min of ischemia; Cr90, creatine + 90 min of ischemia; W180, control/water + 180 min of ischemia; Cr180, creatine + 180 min of ischemia. Green circle, mononuclear cells; blue arrow, polymorphonuclear cells (H.E., 400×).

The number of mononuclear and polymorphonuclear cells in the lung tissue and the perivascular edema index was lower in creatine-treated animals compared to those in the control groups (Figure 3A–C or Figure 4). The illustrative photomicrograph of perivascular edema is shown in Figure 3D.

### 3.6. Proliferation, Apoptosis, and Immune Response

**Figure 5:**
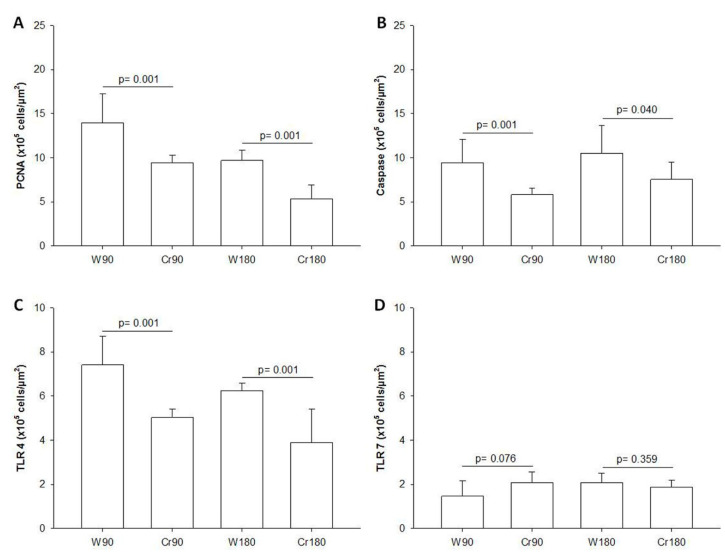
Proliferation, apoptosis and toll-like receptors 4 and 7 in the lung parenchyma. W90, control/water + 90 min of ischemia; Cr90, creatine + 90 min of ischemia; W180, control/water + 180 min of ischemia; Cr180, creatine + 180 min of ischemia. (**A**) Proliferation; (**B**) apoptosis; (**C**) toll-like receptor 4; (**D**) toll-like receptor 7. Data are expressed as mean and standard deviation (*n* = 8 animals/group).

**Figure 6:**
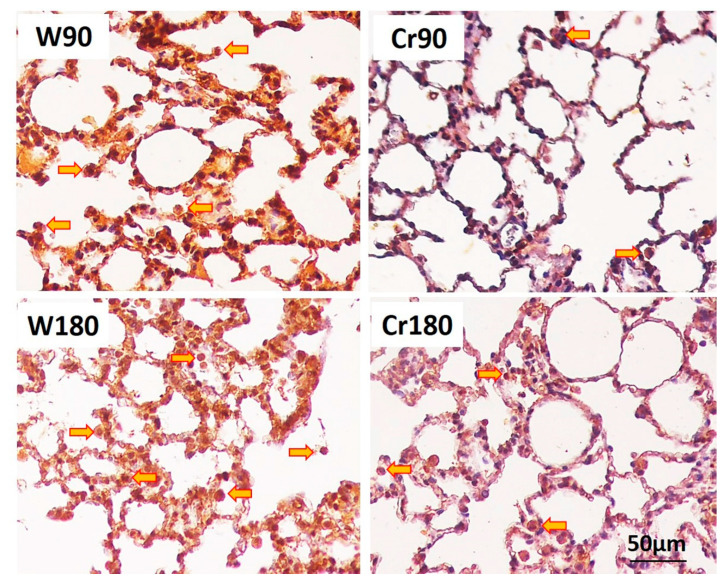
Apoptosis cells in the lung tissue. W90, control/water + 90 min of ischemia; Cr90, creatine + 90 min of ischemia; W180, control/water + 180 min of ischemia; Cr180, creatine + 180 min of ischemia. Orange arrow, macrophages and neutrophils. Photomicrographs of the lung parenchyma stained by Caspase-3 immunohistochemistry (400×).

Creatine-treated animals at the two ischemia times had decreased proliferation, apoptosis, and TLR-4 expression of macrophages and neutrophils in the lung parenchyma. There was no change in TLR-7 expression (Figure 5 and Figure 6).

### 3.7. Levels of Inflammatory Mediators in BALF

**Figure 7:**
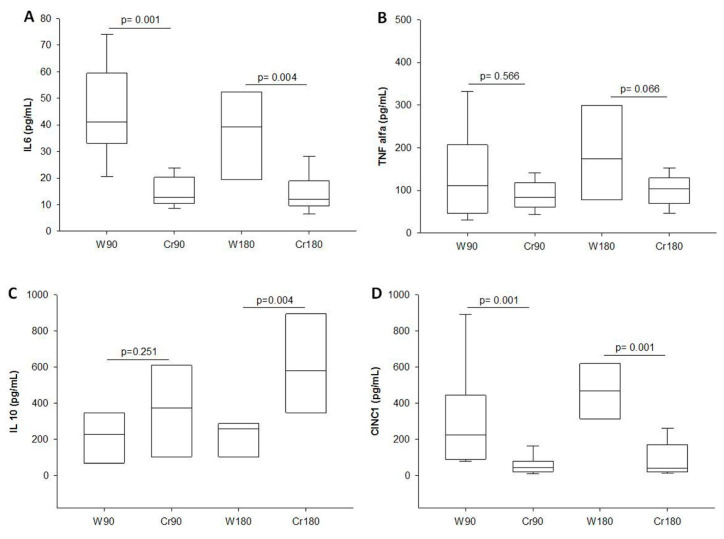
Pro and anti-inflammatory cytokines in the BALF (pg/mL). W90, control/water + 90 min of ischemia; Cr90, creatine + 90 min of ischemia; W180, control/water + 180 min of ischemia; Cr180, creatine + 180 min of ischemia. (**A**) Interleukin 6; (**B**) tumor necrosis factor-alpha; (**C**) interleukin 10; (**D**) cytokine-induced neutrophil chemoattractant 1. Data are expressed as median and interquartile range (*n* = 8 animals/group).

Creatine-treated animals showed lower levels of IL-6 and CINC-1. The IL-10 level was higher in creatine-treated animals after 180 min of ischemia (Figure 7A–D).

## 4. Discussion

Cr supplementation in donor animals for five days before LTx attenuated the effects of IRI. The main findings in the Cr groups were an improvement in pulmonary function and oxygenation as well as a decrease in inflammatory cells in peripheral blood, BALF, and lung parenchyma. Additionally, Cr appears to promote the regulation of inflammatory mediators and immune system.

Although further studies are needed to establish the causality of these findings, several previous studies provide evidence that energetic changes and deregulation of the creatine/creatine kinase (Cr/CK) pathway are closely linked to the etiology of hypoxic disorders and inflammatory drugs [17,18]. All isoenzymes catalyze the reversible transfer of gamma-phosphate from ATP to the guanidine group of Cr to generate phosphocreatine and ADP, thereby mediating efficient storage in the cytosol of high-energy phosphates for rapid and focal replenishment of ATP [18,19,20].

Clinical studies highlight the neuroprotective properties of Cr and the beneficial effects of phosphocreatine on the attenuation of cardiovascular stress [18]. Therefore, we believe that the inclusion of Cr in the diet could be a promising candidate for prophylactic treatment or as a complement to conventional therapies for ischemic diseases or even surgical procedures that require temporary organ ischemia, such as organ transplantation.

IRI is the main cause of PGD and it is associated with the high morbidity and mortality rate of recipient patients in the first days after LTx. Several structural and functional changes modify the integrity of the alveolar-capillary barrier and cause intra-alveolar and interstitial edema [21]. In contrast, IRI is attenuated by the reduced expression of chemokines and proinflammatory cytokines and a decrease in alveolar macrophages [22].

After LTx the majority of alveolar macrophages in the allograft are donor-derived. Donor-derived alveolar macrophages are predominant for at least 2 to 3 years after LTx [23,24]. The early resident leukocyte responses are likely to play a significant role in “initiating” IRI during LTx, and represent potentially important therapeutic targets for reducing PGD [25]. Macrophages maintain high amounts of intracellular creatine. Ji et al. showed that through transporter Slc6a8-mediated uptake, macrophages accumulate high amounts of intracellular creatine that reprogram their polarization by inhibiting IFN-γ while promoting IL-4 [26]. Our study on experimental lungs points to an important role for creatine treatment in donor lungs. Cr supplementation probably improves the conditions against IRI, and studies at the molecular level are required to prove our findings.

In the period of ischemia, the pulmonary parenchyma cells release chemotactic substances [27], resulting in massive adhesion of inflammatory cells connected to arterioles, venules, and alveolar capillaries. The accumulation of red blood cells in IRI is not fully understood [27]. Wolf et al. studied a model of anoxia/reoxygenation in the lung in normothermia and showed a significant increase in the accumulation of red blood cells in the lung [28]. Eppinger et al. showed the accumulation of red blood cells in the non-ischemic collateral lung after 30 min of reperfusion, and suggested the role of chemotactic signaling from the ischemic lung reperfused to the non-ischemic lung [29]. This process of vascular congestion can result in acute edema and impairment of the gas exchange function at the alveolar-capillary membrane level. We observed an improvement in oxygen and carbon dioxide levels in the samples collected from creatine-treated animals.

After reperfusion, pulmonary vascular resistance can also be increased up to three times in relation to normal levels due to vasoconstriction of the pre-capillary pulmonary system after lung IRI [30]. Moreover, increased pulmonary vascular resistance in association with increased vascular permeability [31] results in pulmonary edema in the ischemic [32] and reperfusion periods [33]. As a result, the increase in total and extravascular water content in the lung causes poor gas exchange and worsening of lung mechanics, which leads to low pO2 [33], increased peak airway pressure, and a high alveolar-arterial oxygen gradient [27].

Our results showed the attenuation of vascular edema in creatine-treated animals. Previously, we demonstrated that Cr was able to decrease the deleterious effects of IRI on pulmonary mechanics and edema formation [8]. Moreover, there was an improvement in lung tissue resistance and elastance in animals supplemented with Cr. However, in the present study, these beneficial effects of Cr on pulmonary mechanics were partially observed. Creatine-treated lung function was preserved in immediate reperfusion, but after 2 h of reperfusion, only tissue elastance decreased in both Cr groups (90 and 180 min). We believe that the absence of difference in other parameters in lung mechanics, as observed in the previous study, is due to the fact that the unilateral LTx model is much more complex than the IRI model. For example, this model implies several physiological interactions between the graft and new body. In addition, the graft remained in cold ischemia for 90 or 180 min, where it is not free of damage to cells and tissues.

Apoptosis is a process of programmed cell death during which cells retain membrane integrity and do not release danger-associated molecular patterns (DAMPs), however, these apoptotic cells actively produce anti-inflammatory signals [34]. According to Lockinger et al. [30], after cold ischemia, apoptosis is only found during reperfusion and influenced by the duration of cold ischemia. A moderate time of cold ischemia of 6–12 h before reperfusion triggers more apoptosis in the lung tissue than necrosis. However, a longer cold ischemia time (24 h before reperfusion) resulted in necrosis-dominated cell death [27]. In our study, in which the animals remained in ischemia for 90 or 180 min, increased apoptosis (as denoted by increased caspase-3 expression) and alveolar proliferation of macrophages/neutrophils in lung parenchyma (as denoted by increased density of these cells) were observed at both times in control animals, indicating that damage occurs even within shorter periods of time. Furthermore, the Cr group showed attenuation of this cellular damage.

Innate-immune cells such as macrophages and neutrophils are pivotal in the pathogenesis of IRI. Some studies have documented that, in addition to adaptive immunity, innate immune cells play an important role in transplantation rejection, as allograft loss [34]. IRI is attenuated when alveolar macrophages are reduced, which occurs due to reduced expression of proinflammatory cytokines and chemokines. Reports point to the fundamental role of alveolar macrophages as orchestrators of innate immune responses within the lung [22]. The M1 macrophage is a mainly proinflammatory and tissue destructive subset, which is characterized by increased expression of CD86, inducible nitric oxide synthase (iNOS), TNF-α, IL-1, and IL-6. On the other hand, the M2 macrophage is the anti-inflammatory and tissue-repairing subset, which is characterized by high expression of CD163, CD206, Arg1, and IL-1028. In the present study, we could not perform such analysis, however, it will be addressed in further studies.

The production of TNF-α by alveolar macrophages increases the secretion of proinflammatory cytokines and chemokines by alveolar epithelial cells [35]. The initial phase of IRI is neutrophil independent and characterized by a predominance of TNF-α and IL-1β, whereas the late phase is dependent on recruitment and activation of neutrophils and characterized by increased vascular permeability, chemokines and heterogeneous cytokines [36]. In our study, there was no change in TNF-α in creatine-treated animals. If we relate these data to inflammation of lung tissue and BALF, we can observe that, in our model, there was inflammation mediated by the increase in mononuclear/macrophages cells. We believe that more studies are needed to investigate the production pathway of proinflammatory interleukins in the LTx model in rats.

IL-6 is produced by innumerable cells, including bronchial and lung epithelial cells, whereas IL-10 is released primarily by T-helper type 2 cells. The high level of these two markers means an increased inflammatory state and may have potential for prognosis [37]. Strieter et al. (2002) found that IL-10 gene expression increases when proinflammatory cytokines are regulated in acute inflammatory responses [38]. In our model, we observed a decrease in IL-6 production and increase in IL-10 production in the creatine groups, which suggests an equilibrium in the regulation of interleukins in cell protection. This can also be observed due to decreased CINC-1 production.

The innate immune system recognizes a wide variety of pathogens such as viruses, bacteria, and fungi by standard recognition receptors. These receptors may be connected to the membrane, such as the TLRs, which are expressed by a variety of immune cells including macrophages, monocytes, dendritic cells, and neutrophils [39]. Inflammatory stimuli have been associated with increased expression and activation of TLRs [40]. In our study, there was a decrease in TLR-4 expression in both Cr groups and a tendency to increase TLR-7 expression in 90 min in the Cr group. As in our previous study [8], Cr was able to modulate innate immune system response. The role of TLRs has been investigated in organ transplants and especially with regard to IRI, acute and chronic rejection, and infection [41]. However, the exact role of TLRs in organ transplantation is not fully understood.

Another parameter that is indicative of the inflammatory process is the NOex level. NO is a biological mediator produced by various cell types, including vascular endothelium. It is also an inhibitor of platelet aggregation and neutrophil adhesion and modulates vascular permeability. Additionally, NO acts as a bronchodilator and neurotransmitter [42]. Previous studies have shown that NOex can be used as an indirect marker of pulmonary inflammation and is correlated with severity and response to treatment [43]. In addition, NO is a highly reactive free radical gas that reacts with a wide variety of biomolecules to produce reactive nitrogen species [44].

Almeida et al. [8] showed that Cr is able to ameliorate oxidative damage caused by pulmonary IRI due to the decrease in NOex levels. In this study, with the application of Cr in the IRI model in LTx, we also observed a reduction in oxidative damage at the different ischemia times. Despite understanding the role of oxidants in inflammatory progression, treatments that use a variety of antioxidant approaches have not yet been successful. The failure of some antioxidant clinical trials indicates a gap in our general understanding of whether oxidative stress is beneficial or prejudicial in inflammation [39,45]. More studies that use these antioxidant agents in several experimental models are needed in order to assert their efficacy in simple models of inflammation or more complex models such as IRI in LTx.

New preclinical studies should be conducted to test the best moment, best way and best dosage of Cr administration for improving IRI. For example, Cr could be also used in the graft preservation solution for either flushing or cold storage before the surgical procedure, or it could be administered as an adjuvant therapy for recipients in the postoperative period. Besides, considering species to specific differences, other animal models should be tested in new studies. Based upon the beneficial effects of Cr in attenuating the inflammatory cells influx after IR and in promoting cellular homeostasis presented in this study and previous ones, clinical studies investigating the effects of Cr supplementation are guaranteed.

## 5. Conclusions

In conclusion, Cr supplementation in rats submitted to unilateral pulmonary transplantation attenuated the deleterious effects caused by IRI, as observed through a reduction in inflammation and the preservation of the structure and function of the lung tissue.

## Figures and Tables

**Table 1:** Lung and heart weight.

Weight	W 90 Mean (±SD)	Cr 90 Mean (±SD)	*p*	W 180 Mean (±SD)	Cr 180 Mean (±SD)	*p*
Animal (g)	395.6 (±38.6)	397.6 (±35.7)	0.869	390.4 (±55.5)	394.5 (±32.3)	0.884
Lung (mg)	3040.7 (±732.9)	3064.3 (±733.3)	0.930	3176.8 (±378.8)	3069 (±474.1)	0.540
Heart (mg)	1203.0 (±99.8)	1212.9 (± 119.0)	0.807	1216.5 (±103.8)	1202.7 (±101.3)	0.724

W90, control/water + 90 min of ischemia; Cr90, creatine + 90 min of ischemia; W180, control/water + 180 min of ischemia; Cr180, creatine + 180 min of ischemia. Data are expressed as mean and standard deviation.

**Table 2:** Lung mechanics.

Lung Mechanics	W 90 Median (25–75%)	Cr 90 Median (25–75%)	*p*	W 180 Median (25–75%)	Cr 180 Median (25–75%)	*p*
Immediate reperfusion						
RAW (cmH_2_O.s/mL)	0.071 (0.06–0.11)	0.089 (0.08–0.12)	0.009 *	0.089 (0.07–0.10)	0.101 (0.08–0.11)	0.044 *
GTIS (cmH_2_O/mL)	0.329 (0.31–0.37)	0.322 (0.29–0.33)	0.038 *	0.372 (0.31–0.38)	0.299 (0.27–0.31)	0.001 *
HTIS (cmH_2_O/mL)	1.974 (1.80–2.04)	1.650 (1.56–1.80)	0.003 *	1.881 (1.66–2.31)	1.717 (1.58–1.84)	0.021 *
Final reperfusion						
RAW (cmH_2_O.s/mL)	0.098 (0.07–0.15)	0.092 (0.08–0.12)	0.674	0.096 (0.07–0.15)	0.103 (0.09–0.11)	0.554
GTIS (cmH_2_O/mL)	0.359 (0.32–0.41)	0.325 (0.29–0.35)	0.060	0.361 (0.35–0.37)	0.334 (0.29–0.35)	0.003 *
HTIS (cmH_2_O/mL)	2.312 (1.79–3.11)	1.741 (1.60–2.32)	0.021 *	2.033 (1.69–2.51)	1.739 (1.63–1.85)	0.049 *

W90, control/water + 90 min of ischemia; Cr90, creatine + 90 min of ischemia; W180, control/water + 180 min of ischemia; Cr180, creatine + 180 min of ischemia. RAW, airway resistance; GTIS, tissue damping; HTIS, tissue elastance; * W90 vs. Cr90 and W180 vs. Cr180. Data expressed as median and interquartile range (*n* = 8 animals/group).

**Table 3:** Creatinine and blood gases concentration.

	W 90 Mean (±SD)	Cr 90 Mean (±SD)	*p*	W 180 Mean (±SD)	Cr 180 Mean (±SD)	*p*
Plasma Creatinine (mg/dL)	0.76 (±0.04)	0.84 (±0.07)	0.011 *	0.74 (±0.07)	0.85 (±0.06)	0.011 *
Blood gas						
*p*CO_2_ (mmHg)	32.38 (±12.06)	17.97 (±8.79)	0.006 *	31.37 (±7.06)	24.67 (±7.67)	0.075
*p*O_2_ (mmHg)	74.18 (±23.73)	113.70 (±25.02)	0.002 *	58.85 (±33.42)	115.62 (±24.86)	0.001 *
cLactate (mmol/dL)	7.33 (±1.85)	8.08 (±2.38)	0.459	6.12 (±2.60)	8.74 (±3.19)	0.080

W90, control/water + 90 min of ischemia; Cr90, creatine + 90 min of ischemia; W180, control/water + 180 min of ischemia; Cr180, creatine + 180 min of ischemia. Data are expressed as mean and standard deviation. * W90 vs. Cr90 and W180 vs. Cr180 (*n* = 8 animals/group).

**Table 4:** Peripheral blood and bronchoalveolar lavage fluid (BALF) cells.

Inflammatory Cells	W 90 Mean (±SD)	Cr 90 Mean (±SD)	*p*	W 180 Mean (±SD)	Cr 180 Mean (±SD)	*p*
Peripheral blood cells (×10^4^ cells/mL)						
Total cells	122.25 (±42.82)	64.75 (±32.47)	0.003 *	170.35 (±46.89)	63.75 (±16.17)	0.001 *
Neutrophils	51.51 (±26.85)	25.79 (±15.10)	0.021 *	78.16 (±47.94)	23.98 (±9.74)	0.022 *
Monocytes	21.89 (±10.58)	11.48 (±7.81)	0.017 *	26.72 (±7.34)	10.88 (±4.93)	0.001 *
Lymphocytes	47.64 (±25.31)	26.85 (±13.71)	0.076	64.50 (±18.82)	28.24 (±9.27)	0.001 *
BALF cells (×10^4^ cells/mL)						
Total cells	122.77 (±58.42)	49.44 (±10.13)	0.001 *	133.12 (±75.06)	49.44 (±14.88)	0.001 *
Neutrophils	11.32 (±10.78)	3.86 (±1.91)	0.017 *	17.85 (±11.82)	4.25 (±2.34)	0.001 *
Macrophages	85.19 (±48.84)	33.22 (±7.60)	0.001 *	79.34 (±42.37)	26.96 (±12.23)	0.002 *
Lymphocytes	14.22 (±13.82)	5.09 (±4.39)	0.112	22.79 (±24.62)	12.68 (±6.42)	0.810

W90, control/water + 90 min of ischemia; Cr90, creatine + 90 min of ischemia; W180, control/water + 180 min of ischemia; Cr180, creatine + 180 min of ischemia. Data are expressed as mean and standard deviation. * W90 vs. Cr90 and W180 vs. Cr180 (*n* = 8 animals/group).

## Appendix A

**Table A1:** Donors’ creatinine concentration and lung mechanics.

	W 90 Mean (±SD)	Cr 90 Mean (±SD)	*p*	W 180 Mean (±SD)	Cr 180 Mean (±SD)	*p*
Plasma Creatinine (mg/dL)	0.37 (±0.04)	0.58 (±0.08)	0.002 *	0.39 (±0.02)	0.54 (±0.17)	0.008 *
Lung mechanics						
RAW (cmH2O.s/mL)	0.084 (±0.02)	0.071 (±0.02)	0.129	0.083 (±0.02)	0.080 (±0.01)	0.463
GTIS (cmH2O/mL)	0.234 (±0.04)	0.209 (±0.04)	0.123	0.194 (±0.05)	0.215 (±0.03)	0.242
HTIS (cmH2O/mL)	1.442 (±0.15)	1.359 (±0.09)	0.192	1.440 (±0.09)	1.367 (±0.09)	0.062

W90, control/water + 90 min of ischemia; Cr90, creatine + 90 min of ischemia; W180, control/water + 180 min of ischemia; Cr180, creatine + 180 min of ischemia. Data are expressed as mean and standard deviation. * W90 vs. Cr90 and W180 vs. Cr180 (*n* = 8 animals/group).
